# Acetyl­ene–ammonia–18-crown-6 (1/2/1)

**DOI:** 10.1107/S1600536812038792

**Published:** 2012-09-15

**Authors:** Tobias Grassl, Markus Hamberger, Nikolaus Korber

**Affiliations:** a Institut für Anorganische Chemie, Universität Regensburg, Universitätsstrasse 31, 93053 Regensburg, Germany

## Abstract

The title compound, C_2_H_2_·C_12_H_24_O_6_·2NH_3_, was formed by co-crystallization of 18-crown-6 and acetyl­ene in liquid ammonia. The 18-crown-6 mol­ecule has threefold rotoinversion symmetry. The acteylene mol­ecule lies on the threefold axis and the whole mol­ecule is generated by an inversion center. The two ammonia mol­ecules are also located on the threefold axis and are related by inversion symmetry. In the crystal, the ammonia mol­ecules are located below and above the crown ether plane and are connected by inter­molecular N—H⋯O hydrogen bonds. The acetyl­ene mol­ecules are additionally linked by weak C—H⋯N inter­actions into chains that propagate in the direction of the crystallographic *c* axis. The 18-crown-6 mol­ecule [occupancy ratio 0.830 (4):0.170 (4)] is disordered and was refined using a split model.

## Related literature

For weak inter­molecular inter­actions such as hydrogen bonds and their application in crystal engineering, see: Desiraju (2002 ▶, 2007 ▶); Boese *et al.* (2003 ▶, 2009 ▶); Kirchner *et al.* (2004 ▶); Steiner (2002 ▶)
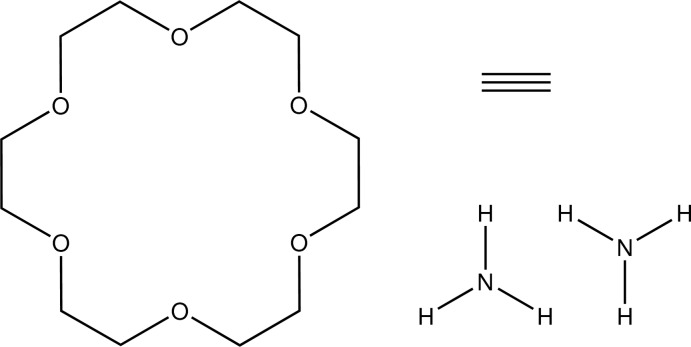


## Experimental

### 

#### Crystal data

C_2_H_2_·C_12_H_24_O_6_·2NH_3_
*M* *_r_* = 324.42
Trigonal, 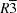

*a* = 11.8915 (1) Å
*c* = 11.5736 (2) Å
*V* = 1417.33 (3) Å^3^
*Z* = 3
Cu *K*α radiation
μ = 0.73 mm^−1^
*T* = 123 K
0.1 × 0.1 × 0.1 mm

#### Data collection

Oxford Diffraction SuperNova diffractometer
Absorption correction: analytical [*CrysAlis PRO* (Agilent, 2012 ▶), based on expressions derived by Clark & Reid (1995 ▶)] *T*_min_ = 0.798, *T*_max_ = 0.841
5835 measured reflections
640 independent reflections
598 reflections with *I* > 2σ(*I*)
*R*_int_ = 0.032

#### Refinement

*R*[*F* ^2^ > 2σ(*F* ^2^)] = 0.036
*wR*(*F* ^2^) = 0.100
*S* = 1.11
640 reflections
53 parameters
H-atom parameters constrained
Δρ_max_ = 0.18 e Å^−3^
Δρ_min_ = −0.19 e Å^−3^

### 

Data collection: *CrysAlis PRO* (Agilent, 2012 ▶); cell refinement: *CrysAlis PRO*; data reduction: *CrysAlis PRO*; program(s) used to solve structure: *olex2.solve* (Bourhis *et al.*, 2012 ▶); program(s) used to refine structure: *SHELXL97* (Sheldrick, 2008 ▶); molecular graphics: *DIAMOND* (Brandenburg & Putz, H, 2011 ▶); software used to prepare material for publication: *OLEX2* (Dolomanov *et al.*, 2009 ▶).

## Supplementary Material

Crystal structure: contains datablock(s) I, global. DOI: 10.1107/S1600536812038792/nc2288sup1.cif

Structure factors: contains datablock(s) I. DOI: 10.1107/S1600536812038792/nc2288Isup2.hkl

Supplementary material file. DOI: 10.1107/S1600536812038792/nc2288Isup3.mol

Supplementary material file. DOI: 10.1107/S1600536812038792/nc2288Isup4.cml

Additional supplementary materials:  crystallographic information; 3D view; checkCIF report

## Figures and Tables

**Table 1:** Hydrogen-bond geometry (Å, °)

*D*—H⋯*A*	*D*—H	H⋯*A*	*D*⋯*A*	*D*—H⋯*A*
N1—H1*A*⋯O1	0.88	2.40	3.2709 (12)	171
N1—H1*A*⋯O1*A*	0.88	2.43	3.270 (4)	159
C1—H1⋯N1	0.95	2.34	3.292 (2)	180

**Fig. 1.**
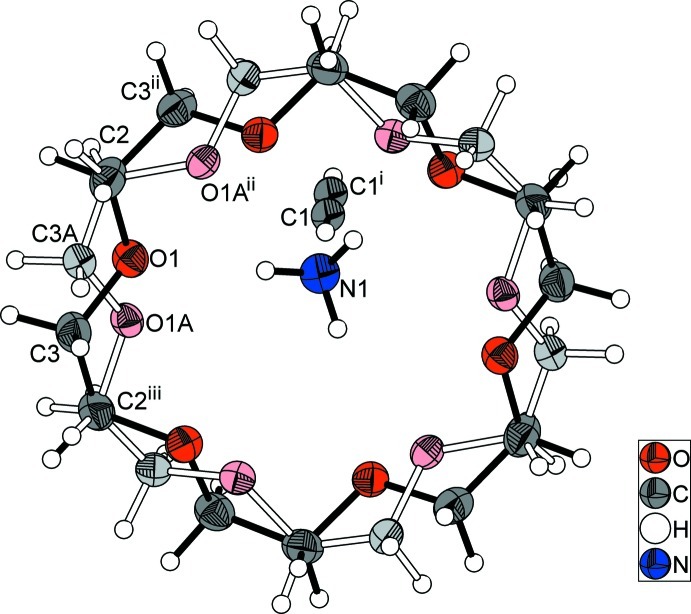
: Crystal structure of the title compound with labeling and displacement ellipsoids drawn at the 50% probability level. Disordering is shown as full and open bonds. Symmetry codes: (i) 2/3 - x, 4/3 - y, 1/3 - z; (ii) -1/3 + y, 1/3 - x + y, 4/3 - z; (iii) 2/3 + x-y, 1/3 + x, 4/3 - z.

**Fig. 2.**
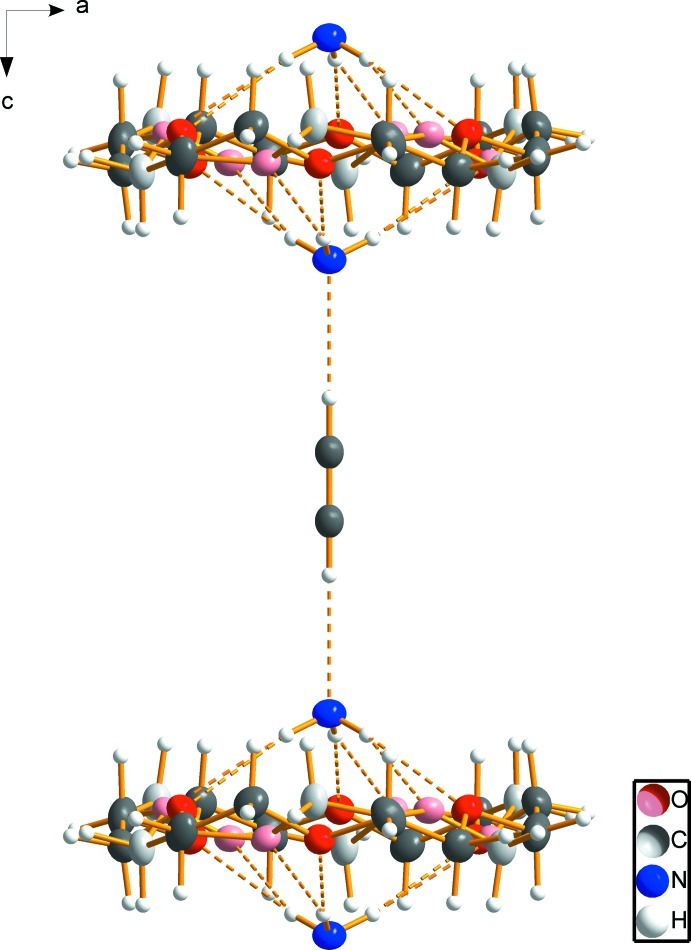
: Crystal structure with view along the b-axis showing the hydrogen bonding interactions. Displacement ellipsoids are drawn at the 50% probability level and disorder is shown as full and open bonds.

**Fig. 3.**
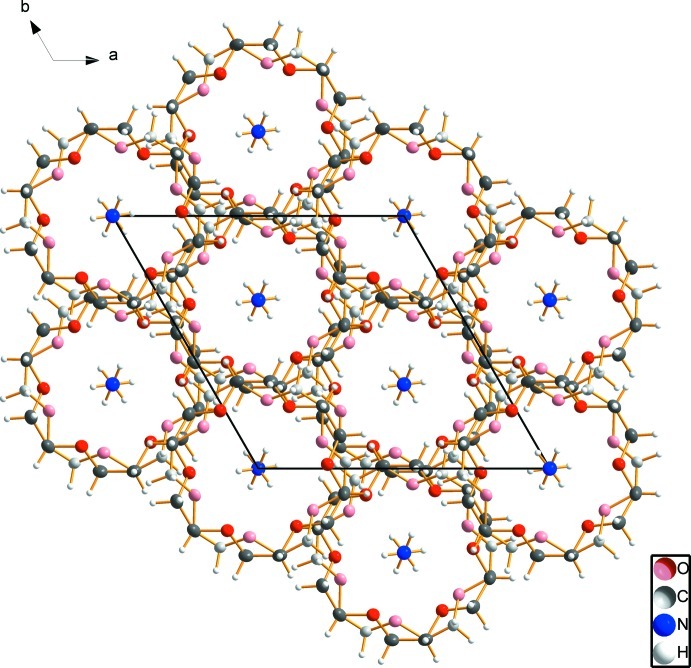
: Projection of the unit cell along the crystallographic c-axis. Displacement ellipsoids are drawn at the 50% probability level and disorder is shown as full and open bonds

## supplementary crystallographic information

### Comment

The crystal structure of the title compound was determined in the course of investigations regarding the reactivity of acetylene in liquid ammonia.

In the crystal structure the acetylene molecule shows moderate hydrogen bonding in axial direction to an ammonia molecule on each side with a H···N distance of 2.3422 (15) Å and a C—H···N angle of 180°. Two ammonia molecules are located below and above the crown ether plane, bound by hydrogen bonds to the oxygen atoms in the ring (Fig. 1 and Fig. 2). Both ammonia molecules are connected to 18-crown-6 *via* three hydrogen bonds each with a H···O distance of 2.40 Å and a N—H···O angle of 171.0° for the crown ether part with a site occupation factor of 0.83 and with a H···O distance of 2.43 Å and a N—H···O angle of 159.4° for the crown ether part with a site occupation factor of 0.17 (Fig. 2 and Table 1).This arrangement leads to one-dimensional strands along the crystallographic *c*-axis, that are packed in a kind of hexagonal closest arrangement (Fig. 3). The formation of hydrogen bonds between acetylene and ammonia molecules as well as the interaction of ammonia molecules with the crown ether is essential to stabilize the fugitive acetylene molecule in the solid state as was shown previously by Boese *et al.* (Boese *et al.*, 2009) in C_2_H_2_*NH_3_. Due to the absence of stronger intermolecular interactions the optimization of hydrogen bonds is the driving force for the axial stacking of the molecules along the crystallographic *c*-axis. This can also be observed in acetylene containing material such as co-crystallized C_2_H_2_*NH_3_ (Boese *et al.*, 2009) and co-crystals of acetylene and acetone/DMSO (Boese *et al.*, 2003) or azacycles (Kirchner *et al.*, 2004).

### Experimental

0.039 g(1.0 mmol) potassium and 0.264 g(1.00 mmol)18-crown-6 were placed under argon atmosphere in a baked-out reaction vessel and 30 ml of dry liquid ammonia were condensed. The mixture was stored at 236 K for one week to ensure that all substances were completely dissolved. Afterwards an excess of acetylene gas was fed into the solution until the colour changed from deep blue to colourless. Colourless crystals of the title compound were obtained after further storage at 236 K for nine month. Well soluble potassium hydrogen acetylide KC_2_H remained in solution.

### Refinement

The O atom and one C atom of the crown ether are disordered and were refined using a split model with sof of 0.830 (4) and 0.170 (4). The C—H H atoms were positioned with idealized geometry and refined isotropic with *U*_iso_(H) = 1.2 *U*_eq_(C) using a riding model. The N-H H atom was located in difference map and refined in the riding mode approximation.

### Crystal data

**Table:**

C_2_H_2_·C_12_H_24_O_6_·2NH_3_	*D*_x_ = 1.140 Mg m^−^^3^
*M**_r_* = 324.42	Cu *K*α radiation, λ = 1.54184 Å
Trigonal, *R*3	Cell parameters from 3585 reflections
Hall symbol: -R 3	θ = 5.8–73.3°
*a* = 11.8915 (1) Å	µ = 0.73 mm^−^^1^
*c* = 11.5736 (2) Å	*T* = 123 K
*V* = 1417.33 (3) Å^3^	Block, clear colourless
*Z* = 3	0.1 × 0.1 × 0.1 mm
*F*(000) = 534	

### Data collection

**Table:**

Oxford Diffraction SuperNova diffractometer	640 independent reflections
Radiation source: fine-focus sealed tube	598 reflections with *I* > 2σ(*I*)
Graphite monochromator	*R*_int_ = 0.032
ω scans	θ_max_ = 73.3°, θ_min_ = 5.8°
Absorption correction: analytical [*CrysAlis PRO* (Agilent, 2012), based on expressions derived by Clark & Reid (1995)]	*h* = −14→14
*T*_min_ = 0.798, *T*_max_ = 0.841	*k* = −14→14
5835 measured reflections	*l* = −14→14

### Refinement

**Table:**

Refinement on *F*^2^	Primary atom site location: iterative
Least-squares matrix: full	Secondary atom site location: difference Fourier map
*R*[*F*^2^ > 2σ(*F*^2^)] = 0.036	Hydrogen site location: inferred from neighbouring sites
*wR*(*F*^2^) = 0.100	H-atom parameters constrained
*S* = 1.11	*w* = 1/[σ^2^(*F*_o_^2^) + (0.0481*P*)^2^ + 0.8298*P*] where *P* = (*F*_o_^2^ + 2*F*_c_^2^)/3
640 reflections	(Δ/σ)_max_ < 0.001
53 parameters	Δρ_max_ = 0.18 e Å^−^^3^
0 restraints	Δρ_min_ = −0.19 e Å^−^^3^

### Special details

**Table:**

Experimental. Absorption correction: Crysalis Pro, Agilent Technologies, Version 1.171.35.21, Analytical numeric absorption correction using a multifaceted crystal model based on expressions derived by R. C. Clark & J. S. Reid (1995).Crystal mounting in perfluorether
Geometry. All e.s.d.'s (except the e.s.d. in the dihedral angle between two l.s. planes) are estimated using the full covariance matrix. The cell e.s.d.'s are taken into account individually in the estimation of e.s.d.'s in distances, angles and torsion angles; correlations between e.s.d.'s in cell parameters are only used when they are defined by crystal symmetry. An approximate (isotropic) treatment of cell e.s.d.'s is used for estimating e.s.d.'s involving l.s. planes.
Refinement. Refinement of *F*^2^ against ALL reflections. The weighted *R*-factor *wR* and goodness of fit *S* are based on *F*^2^, conventional *R*-factors *R* are based on *F*, with *F* set to zero for negative *F*^2^. The threshold expression of *F*^2^ > σ(*F*^2^) is used only for calculating *R*-factors(gt) *etc*. and is not relevant to the choice of reflections for refinement. *R*-factors based on *F*^2^ are statistically about twice as large as those based on *F*, and *R*- factors based on ALL data will be even larger.

### Fractional atomic coordinates and isotropic or equivalent isotropic displacement parameters (Å^2^)

**Table:**

	*x*	*y*	*z*	*U*_iso_*/*U*_eq_	Occ. (<1)
O1	0.34988 (11)	0.43774 (10)	0.64475 (7)	0.0298 (4)	0.830 (4)
C2	0.23519 (11)	0.32279 (10)	0.67833 (11)	0.0387 (4)	
H2AA	0.2314	0.3164	0.7637	0.046*	0.830 (4)
H2AB	0.2375	0.2463	0.6476	0.046*	0.830 (4)
H2BC	0.1912	0.2473	0.7309	0.046*	0.170 (4)
H2BD	0.2225	0.2880	0.5987	0.046*	0.170 (4)
C3	0.46325 (13)	0.45185 (13)	0.69816 (12)	0.0344 (4)	0.830 (4)
H3B	0.4711	0.3743	0.6819	0.041*	0.830 (4)
H3A	0.4568	0.4586	0.7829	0.041*	0.830 (4)
O1A	0.4390 (5)	0.5013 (5)	0.6463 (3)	0.0254 (17)	0.170 (4)
C3A	0.3587 (6)	0.3803 (6)	0.7006 (6)	0.0312 (12)	0.17
H3AB	0.3905	0.3206	0.6786	0.037*	0.170 (4)
H3AA	0.3700	0.3934	0.7852	0.037*	0.170 (4)
N1	0.3333	0.6667	0.50242 (13)	0.0330 (4)	
H1A	0.3430	0.6046	0.5340	0.040*	
C1	0.3333	0.6667	0.21796 (16)	0.0289 (4)	
H1	0.3333	0.6667	0.3000	0.035*	

### Atomic displacement parameters (Å^2^)

**Table:**

	*U*^11^	*U*^22^	*U*^33^	*U*^12^	*U*^13^	*U*^23^
O1	0.0306 (8)	0.0310 (6)	0.0287 (5)	0.0160 (5)	0.0010 (4)	0.0042 (4)
C2	0.0397 (7)	0.0276 (6)	0.0487 (7)	0.0168 (5)	0.0111 (5)	0.0046 (4)
C3	0.0379 (8)	0.0347 (7)	0.0359 (8)	0.0220 (7)	−0.0045 (5)	−0.0002 (5)
O1A	0.027 (3)	0.029 (3)	0.022 (2)	0.016 (2)	−0.0035 (16)	0.0004 (17)
C3A	0.027 (3)	0.029 (3)	0.040 (3)	0.016 (3)	−0.002 (2)	0.000 (3)
N1	0.0363 (6)	0.0363 (6)	0.0263 (7)	0.0182 (3)	0.000	0.000
C1	0.0257 (5)	0.0257 (5)	0.0351 (8)	0.0129 (3)	0.000	0.000

### Geometric parameters (Å, º)

**Table:**

O1—C2	1.4196 (15)	C3—H3B	0.9900
O1—C3	1.4148 (18)	C3—H3A	0.9900
C2—H2AA	0.9900	O1A—C2^ii^	1.446 (5)
C2—H2AB	0.9900	O1A—C3A	1.416 (8)
C2—H2BC	0.9900	C3A—H3AB	0.9900
C2—H2BD	0.9900	C3A—H3AA	0.9900
C2—C3^i^	1.4698 (18)	N1—H1A	0.8810
C2—O1A^i^	1.446 (5)	C1—C1^iii^	1.187 (4)
C2—C3A	1.298 (6)	C1—H1	0.9500
C3—C2^ii^	1.4698 (18)		
			
C3—O1—C2	113.22 (10)	C3A—C2—H2BC	107.5
O1—C2—H2AA	109.4	C3A—C2—H2BD	107.5
O1—C2—H2AB	109.4	C3A—C2—C3^i^	152.4 (3)
O1—C2—H2BC	149.2	C3A—C2—O1A^i^	119.3 (3)
O1—C2—H2BD	91.2	O1—C3—C2^ii^	110.55 (11)
O1—C2—C3^i^	111.27 (10)	O1—C3—H3B	109.5
O1—C2—O1A^i^	89.71 (18)	O1—C3—H3A	109.5
H2AA—C2—H2AB	108.0	C2^ii^—C3—H3B	109.5
H2BC—C2—H2BD	107.0	C2^ii^—C3—H3A	109.5
C3^i^—C2—H2AA	109.4	H3B—C3—H3A	108.1
C3^i^—C2—H2AB	109.4	C3A—O1A—C2^ii^	122.5 (4)
C3^i^—C2—H2BC	97.7	C2—C3A—O1A	117.3 (5)
C3^i^—C2—H2BD	74.6	C2—C3A—H3AB	108.0
O1A^i^—C2—H2AA	87.6	C2—C3A—H3AA	108.0
O1A^i^—C2—H2AB	148.6	O1A—C3A—H3AB	108.0
O1A^i^—C2—H2BC	107.5	O1A—C3A—H3AA	108.0
O1A^i^—C2—H2BD	107.5	H3AB—C3A—H3AA	107.2
C3A—C2—H2AA	80.7	C1^iii^—C1—H1	180.0
C3A—C2—H2AB	90.6		

Symmetry codes: (i) *y*−1/3, −*x*+*y*+1/3, −*z*+4/3; (ii) *x*−*y*+2/3, *x*+1/3, −*z*+4/3; (iii) −*x*+2/3, −*y*+4/3, −*z*+1/3.

### Hydrogen-bond geometry (Å, º)

**Table:**

*D*—H···*A*	*D*—H	H···*A*	*D*···*A*	*D*—H···*A*
N1—H1*A*···O1	0.88	2.40	3.2709 (12)	171
N1—H1*A*···O1*A*	0.88	2.43	3.270 (4)	159
C1—H1···N1	0.95	2.34	3.292 (2)	180
